# Gun violence in intimate partner violence: insights from social media

**DOI:** 10.1186/s12889-026-27059-z

**Published:** 2026-03-25

**Authors:** Yiyang Mei, Ayush Kothari, Juliana Upchurch, Sangmi Kim

**Affiliations:** 1 School of Law, Emory University, Atlanta, GA USA; 2 College of Arts & Sciences, Emory University, Atlanta, GA USA; 3 School of Global Public Health, New York University, New York, NY USA; 4 Nell Hodgson Woodruff School of Nursing, Emory University, Atlanta, GA USA

**Keywords:** Gun violence, Firearm, Intimate partner violence, Social media

## Abstract

**Background:**

This study analyzed publicly available Reddit data to characterize gun violence in intimate partner violence (IPV) via quantitative content analysis.

**Methods:**

We examined 105 Reddit posts detailing firsthand and vicarious accounts of firearm-related IPV authored by self-identified survivors (94%) and bystanders (6%), including non-fatal shooting. We extracted data on forms and tactics of IPV, survivors’ needs, firearm-related behaviors, and consequences of these experiences.

**Results:**

We found that Reddit posts contained rich information about firearm use against intimate partners, most often describing non-fatal incidents. Victims whose partners used firearms to threaten or intimidate them were more likely to report experiencing the threat of physical and sexual violence, physical injury, and ongoing safety concerns.

**Conclusions:**

Social media data collected in real-time, anonymously, and unobtrusively can better inform the circumstances around gun violence in IPV and help develop prevention strategies.

## Background

Intimate partner violence (IPV), marked by physical violence, sexual violence, stalking, and psychological aggression (e.g., expressive aggression, coercive control, threat of physical or sexual violence, exploitation of survivors’ vulnerability, and gaslighting), is a critical public health issue in the United States and on a global scale [1]. Victims often describe certain tactics of psychological aggression, such as narcissistic abuse and financial abuse, as stand-alone forms of IPV, reflecting variation in how IPV is defined and categorized [2]. According to the Centers for Disease Control and Prevention (CDC), about 41% of women and 26% of men experienced contact sexual violence, physical violence, or stalking by an intimate partner during their lifetime and reported a related impact [3]. IPV results in tremendous physical, emotional, and social sequelae for both victims and their families [4]. The wide-ranging impacts can also make it more difficult for victims to build healthier relationships in the future and can even lead them to another abusive relationship [5]. Children who witness violence are more likely to either perpetrate or fall victim to violence themselves in the future, resulting in extensive secondary effects and generational trauma [6].

Firearm use is a mark of an escalation in violence. A firearm is often used, fatally or non-fatally, to harm and control intimate partners. Approximately 4.5 million U.S. women have been threatened by an abuser with a firearm; nearly 1 million U.S. women have been shot by an abuser without fatal consequences [7]. Women in the U.S. are most commonly murdered by a current or former intimate partner using a firearm [8]. Firearm access and regulations vary across U.S. states; for instance, California enforces some of the strictest laws, including background checks, whereas Texas maintains comparatively permissive open- and concealed-carry laws and fewer restrictions on firearm ownership—conditions that shape the risk environment for IPV and firearm-related intimate partner homicide (IPH) [9]. Abusers’ access to firearms increases the likelihood of IPV progressing to IPH five-fold [10]. Moreover, being threatened with a firearm can lead to adverse mental health outcomes, such as anxiety, insomnia, and post-traumatic stress disorder [11].

Despite growing recognition of firearm use in IPV, patterns and impacts of firearm-related IPV are not well-understood due to data limitations. Nationally representative surveys, such as the National Intimate Partner and Sexual Violence Survey (NISVS), have not consistently captured firearm use in IPV. Self-reported data – common in IPV research are prone to biases, including underreporting due to embarrassment and concerns about confidentiality [12]. Medical records, though valuable, are often incomplete. Healthcare providers rarely screen for IPV routinely due to various barriers, including general unwillingness, time constraints, lack of training, lack of knowledge of how to help if IPV is disclosed, fear of offending patients, the absence of clear protocols, belief that patients will not disclose, and the presence of the woman’s partner or other family members during the visit [13, 14].​ Similar challenges arise when providers inquire about firearms [15], further limiting the utility of medical records for examining the role of firearms in IPV. Thus, alternative data sources are critical to capturing more accurate insights into firearm-related IPV.

Social media offers an alternative data source for secondary analyses in IPV research. Platforms such as Reddit, known for their anonymity through random usernames and throwaway accounts, provides unique spaces where users can openly discuss sensitive topics, such as mental illness and IPV [2, 16]. IPV survivors often turn to informal networks, such as friends, family, or online communities, for support rather than formal institutions like healthcare providers or law enforcement [17]. In this context, Reddit serves as an anonymous venue where survivors seek informal support, share personal experiences, and crowdsource advice, revealing insights that might never emerge in formal settings.

Despite its potential, Reddit data have notable limitations. The platform’s user base is not representative of the general population—it skews younger, tech-savvy, and primarily male. Nonetheless, Reddit’s large and active community, averaging 2.2 billion monthly visits on average as of March 2024 [18], provides access to diverse, spontaneous discussions about IPV. While national, population-based surveys and qualitative studies have successfully engaged IPV survivors, such research typically relies on structured instruments or institutional recruitment. In contrast, Reddit offers anonymized and unprompted accounts that capture everyday experiences, perceptions, and social attitudes surrounding IPV that may be less visible in formal research contexts [12, 19]. These data thus complement, rather than replace, traditional sources by illuminating how survivors articulate and negotiate their experiences in informal, real-time settings.

Taking all this into account, this study conducts a secondary analysis of Reddit posts to examine firearm use in IPV. Its objectives are to (1) assess the availability of information on firearm-related IPV on Reddit and (2) describe the characteristics of firearm use in IPV as reported on the platform.

## Methods

A secondary analysis was conducted on Reddit data from the parent study that explored IPV-related information shared on the platform [2]. While the parent study provided an overview of IPV narratives, this study specifically focuses on IPV survivors’ accounts of firearm-related violence (or gun violence [GV]) perpetrated by their intimate partners.

### Data collection for IPV

The parent study collected publicly available Reddit posts written in English from four IPV-related subreddits: *r/domesticviolence* (DV), *r/abusiverelationships* (AR), *r/ AbuseInturrpted* (AI), and *r/relationships* (Rel) [2]. Posts were retrieved using the Python Reddit Application Programming Interface (API) Wrapper (PRAW). We searched Reddit using relevant keywords (e.g., “IPV,” “domestic violence,” “abuse,” and “abusive relationships”) and selected these four subreddits that included extensive first-person accounts of IPV and active exchange of information and support [2]. Data collection spanned from January 1, 2020, to March 31, 2021, yielding 4,000 IPV-related Reddit posts. To remove posts with low interactions, only posts with a score of three or higher (# of upvotes - # of downvotes) were retained [2], reducing the dataset to 2,755 posts. These posts, including user IDs, timestamps, and text, were exported to Microsoft Excel for annotation.

### Data extraction for GV

**Fig. 1:**
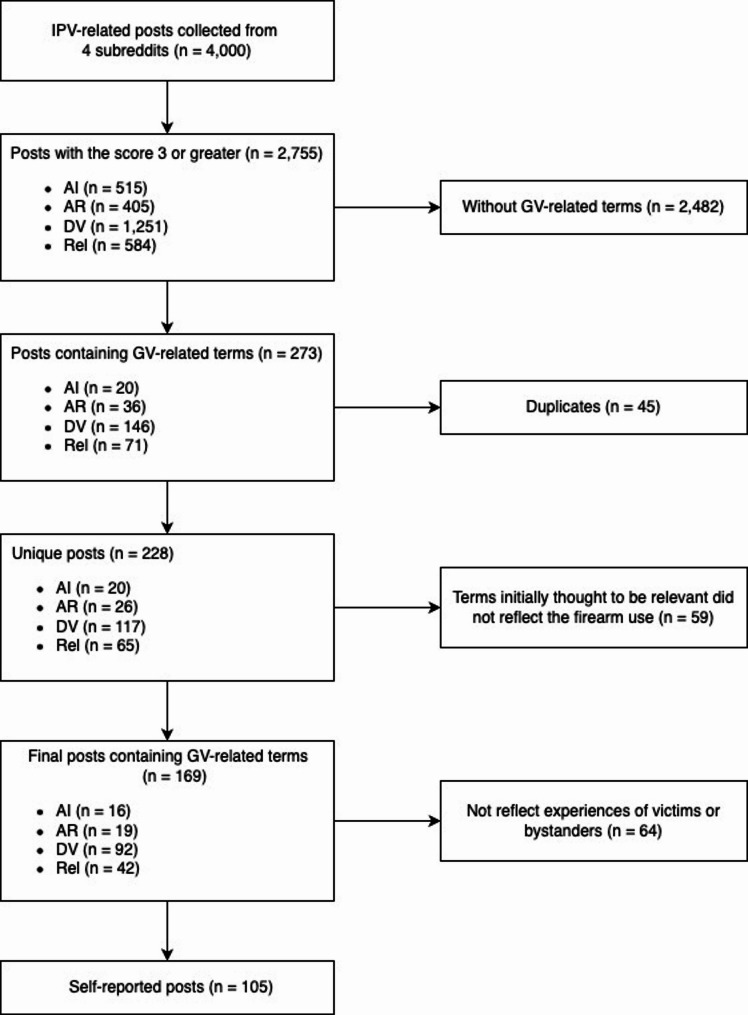
Selection of intimate partner violence and gun violence posts on reddit

From the 2,755 posts, those specifically mentioning firearm-related violence were identified using keywords such as “*gun*,” “*firearm*,” “*pistol*,” “*Glock*,” “*shot*,” “*shoot*,” and “*fire*” (*n* = 273). These keywords were carefully selected by the research team to ensure relevance and capture related terminology (e.g., “*handgun*” via “*gun*”). Duplicate posts were removed using Excel’s Conditional Formatting function, yielding 228 unique posts containing firearm-related keywords. Posts with irrelevant mentions (e.g., “*photoshoot*,” “*wildfire*,”) were manually screened out, resulting in 169 posts (AI: 16, AR: 19, DV: 92, Rel: 42). Posts that did not reflect experiences of survivors or bystanders were then excluded, yielding a final dataset of 105 posts. Only original posts (OPs) were included, excluding reply threads under the OPs (Fig. 1).

To ensure all firearm-related posts were identified, two methods were used. First, manual extraction was conducted as described. Second, an Excel macro was developed to systematize the process by scanning for keywords and assigning relevance scores to posts. For example, figurative uses of terms like “*sticking to my firearms*” were assigned lower relevance. The results from the manual and automated methods were then compared to verify that all relevant posts were captured in the manual extraction.

### Codebook development

A codebook was collaboratively developed by the principal investigator (PI) and two research assistants (RAs) using deductive codes based on the CDC’s “Intimate Partner Violence Surveillance Uniform Definitions and Recommended Data Elements Version 2.0” [20]. The parent study’s codebook included: (a) self-report status of IPV (e.g., self-reported or reported by family/friend/neighbor); (b) timing of IPV (i.e., past or current/ongoing); (c) forms of IPV (i.e., physical violence, sexual violence, stalking, or psychological aggression); (d) tactics of psychological aggression (i.e., expressive aggression, coercive control, a threat of physical or sexual violence, exploitation of a survivor’s vulnerability, or gaslighting); (f) help-seeking status (i.e., not help-seeking or help-seeking for survivor self); (g) survivors’ needs.

Guided by Adhia et al. [21], the research team developed additional codes specific to firearm-related IPV, including: (a) whether a firearm was used, (b) how the firearm was used, (c) common behaviors associated with firearm use, and (d) consequences of IPV and firearm use. Table 1 presents examples (excerpts) of firearm-related IPV codes from the subreddits. Examples of IPV-related codes can be found elsewhere [2].

**Table 1:** Victims’ narrative of firearm use in intimate partner violence on reddit

Code	Segment
How the firearm was used (*N* = 4)	
Threatened by a partner who possessed or had easy access to a firearm	*“He has access to firearms and will hurt my friends at school or work.”*
Threatened with a firearm	*“He liked to hold a gun to my head and make me beg to be slapped around.”*
Had a firearm used on the victim	*“I’ve been stabbed*,* shot at*,* beaten with blunt objects*,* and even beaten while I laid there unconscious from a vicious assault.”*
Used for self-defense from a partner	*“I sleep with my gun in my nightstand.”*
Firearm use behavior (*N* = 9)	
Displayed firearm	*“He would sit with his loaded .45 sitting in his lap or pointed it at me or my head*,* telling me if I ever lied to him again*,* he promised he’d kill me.”*
Threatened to shoot the victim	*“He said he was going to get his gun to kill me.”*
Threatened to shoot the abuser	*“He told me he is going to get his gun and shoot himself in the head.”*
Threatened to shoot someone else	*“He said he’s beating my ass first*,* or he’s going to shoot everyone that lives on my floor of apt.”*
Threatened to shoot a pet	*“I kept getting calls and texts from him*,* and once he started threatening to kill himself and our pets…”*
Shot the firearm but did not hit anyone	*“He loaded the .38 and took the safety off*,* he had already beaten me pretty badly and he took the .38 fired it one time…”*
Hit the victim’s body with the firearm	*“I have been hit in the head with a gun by my ex*,* so I did not take those threats lightly.”*
Shot the victim	*“He had shot me. It burned bad and the searing pain in my side made it hard catch air.”*
Shot the abuser	*“My ex would cut himself in front of me and threaten suicide if I left… He came out with it pointed to his head and shot himself. He collapsed and I felt the overwhelming guilt that I had killed him. Well*,* turns out he shot himself with a blank bullet…”*

The narrative regarding “shot someone else” was not available in the analyzed Reddit data

The PI and two RAs tested the initial codebook by coding five posts independently, resolving discrepancies collaboratively, and refining the definitions. Over time, the codebook evolved to capture new themes emerging from survivors’ narratives. Numeric codes were assigned to each category, such as four codes for how the firearm was used, ten for the common behaviors associated with firearm use, and 20 for the consequences of IPV and firearm use.

### Data annotation and analysis

Using the finalized codebook, two RAs independently annotated the remaining posts, while the PI reviewed and resolved discrepancies. Quantitative content analysis was conducted using words as the coding unit [22]. Due to the small sample size, analyses were primarily descriptive and exploratory. Descriptive statistics (frequency, percentage, and range) were used to characterize IPV and firearm use. Fisher’s exact test, Pearson’s Chi-squared test, and Wilcoxon rank sum test were used to explore differences in IPV patterns and consequences based on abusers’ firearm use, without making inferential claims. All analyses were performed using R (version 4.2.3).

## Results

Table 2 shows the basic characteristics of IPV reported in the 105 OPs across the four subreddits. Nearly half of the OPs (47%) described IPV that victims were currently experiencing. 47% indicated that the victim was female and 8% indicated male. Although the percentage of female victims could be higher than 47%, the victims’ gender was coded only when explicitly mentioned. 46% of the OPs did not specify the victims’ gender, whereas the majority of abusers (88%) were indicated as male. 37% of OPs reported that victims had children, either shared with the abusers or from other relationships, while the remaining 63% did not mention the presence or involvement of children in the violent incident. While multiple forms of violence concurrently occurred, the most reported was psychological aggression (88%), followed by physical violence (71%) and sexual violence (22%). 42% of OPs contained help-seeking messages written by the victims. Although all 105 OPs included terminology potentially related to GV, not every post described the use of a firearm by an intimate partner. Only 30% explicitly reported firearm use against the victim.

**Table 2:** Characteristics of the self-reported intimate partner violence (*n* = 105)

	Overall
	*N* = 105^1^
Reported by	
Victim self	99 (94%)
Bystander	6 (5.7%)
Time of IPV	
Past	56 (53%)
Current/Ongoing	49 (47%)
Gender of Victim	
Male	8 (7.6%)
Female	49 (47%)
Not mentioned	48 (46%)
Gender of Abuser	
Male	92 (88%)
Female	12 (11%)
Not mentioned	1 (1.0%)
Co-Abuse	
Yes	3 (2.9%)
Not mentioned	102 (97%)
Duration of Relationship	
Median (Range)	4.0 (3.0, 6.0)
Not mentioned	60
Have Child(ren)	
Yes	39 (37%)
Not mentioned	66 (63%)
Type of IPV^2^	
Psychological Aggression	92 (88%)
Physical Violence	75 (71%)
Sexual Violence	23 (22%)
Financial Abuse	20 (19%)
Narcissistic Abuse	9 (8.6%)
Not Specified	6 (5.7%)
Stalking	5 (4.8%)
Tactics of Psychological Aggression (*n* = 92)^2,3^	
Expressive Aggression	74 (80%)
Threat of Physical and Sexual Violence	41 (45%)
Coercive Control	38 (41%)
Gaslighting	24 (26%)
Exploitation of Vulnerability	10 (11%)
Not mentioned	13
Seek Help	
No	59 (56%)
Yes (by survivors)	44 (42%)
Yes (by bystanders)	2 (1.9%)
Use of Firearm	
No	74 (70%)
Yes	31 (30%)

The initial sample of 169 was reduced to those self-reporting intimate partner violence (*n* = 105)

^1^ n (%); Median (IQR); Range

^2^ Types and tactics of IPV were not mutually exclusive; a single post could be coded for multiple forms and tactics of IPV, resulting in totals greater than 100%

^3^ The analysis was limited to 92 survivors reporting psychological aggression

When breaking down the psychological aggression (*n* = 92) into different tactics, expressive aggression was the predominant form of psychological aggression (80%), followed by threat of physical and sexual violence, coercive control, gaslighting, and exploitation of vulnerability.

In addition, victims reported a wide range of needs. Of 44 help-seeking OPs, nearly one-third of the posts asked for advice on what they should do in the circumstances they faced. In 14% of the OPs, victims asked for advice on safety or escape planning. One in ten posts also asked about ways to cope, similar experiences, validation of feelings/reactions/thoughts/actions, and clarification on whether they were being abused (Table 3).

**Table 3:** Victims’ needs when seeking help (*n* = 44)

	Overall
	*N* = 44^1^
Ask advice on what to do	12 (27%)
Ask advice on safety or escape planning	6 (14%)
Ask ways to cope	5 (11%)
Ask similar experiences	4 (9.1%)
Ask if my feelings/reactions/thoughts/actions are valid	4 (9.1%)
Want to know if I am being abused	4 (9.1%)
Want legal advice	3 (6.8%)
Ask advice on relationship issues with their abusive current/ex-partners	3 (6.8%)
Want financial advice	3 (6.8%)
Want to move on	2 (4.5%)
Want to help the perpetrator	1 (2.3%)
Ask helpfulness of interventions for perpetrators	1 (2.3%)
Need (referral to) resources for help	1 (2.3%)
Need assurance of self-worth	1 (2.3%)
Want to know why I am experiencing certain symptoms	1 (2.3%)
Try to make sense of what happened	1 (2.3%)
Ask how to address issues in building a new relationship after leaving the past abusive relationship	1 (2.3%)

Out of 25 need categories, eight were excluded because there were no affirmative cases. Such categories include asking if violence can be justified in any circumstances; wanting to educate the perpetrator; asking for information/experience on specific services for victims; wanting to know other options to receive help; wanting to have hope for a better life and relationship; wanting to recover/restore/heal myself; asking ways to grieve; and asking about experience/information regarding emotion/behavior/health/development/coping of the victims’ children

^1^ n (%)

Among IPV victims of GV (*n* = 31), 61% indicated that they had been threatened with a firearm, and 29% indicated having been threatened by a partner who had easy access to a firearm. The most common behavior of firearm use was threatening to shoot the victim (48%), followed by the abuser threatening to shoot themselves (threat to commit suicide) (23%), displaying the firearm (19%), and threatening to shoot someone else (19%), although actual shooting also occurred in a few cases (Table 4). The victims reported experiencing a maximum of three firearm use behaviors at the same time: Of 31 IPV victims of GV, about 45% reported the involvement of one behavior, and 35% two or more.

**Table 4:** Characteristics of firearm use in intimate partner violence (*n* = 31)

	Overall
	*N* = 31^1^
How firearm was used	
Were threatened by a partner who possessed or had easy access to a firearm	9 (29%)
Were threatened with a firearm	19 (61%)
Had a firearm used on the victim (e.g., shot or shot at)	3 (9.7%)
Used for a self-defense from a partner	1 (3.2%)
Common behavior of firearm use	
Displayed firearm	6 (19%)
Threatened to shoot the victim	15 (48%)
Threatened to shoot the abuser	7 (23%)
Threatened to shoot someone else	6 (19%)
Threatened to shoot a pet	0 (0%)
Shot the firearm but did not hit anyone	1 (3.2%)
Hit the victim’s body with a firearm	1 (3.2%)
Shot someone else	0 (0%)
Shot the victim	1 (3.2%)
Shot the abuser	2 (6.5%)

^1^ n (%)

When stratifying the reported IPV patterns by firearm use, IPV victims who experienced GV were more likely to report the threat of physical and sexual violence than their counterparts who did not experience GV (83% vs. 27%, *p* = < 0.001) (Table 5).

**Table 5:** Patterns of intimate partner violence by the use of firearm (*n* = 105)

Characteristic	Overall	Firearm Not Used	Firearm Used	*p*-value^2^
	*N* = 105^1^	*N* = 74^1^	*N* = 31^1^	
Not Specified	6 (5.7%)	5 (6.8%)	1 (3.2%)	0.7
Physical Violence	75 (71%)	52 (70%)	23 (74%)	0.7
Sexual Violence	23 (22%)	16 (22%)	7 (23%)	> 0.9
Stalking	5 (4.8%)	5 (6.8%)	0 (0%)	0.3
Psychological Aggression	92 (88%)	63 (85%)	29 (94%)	0.3
Narcissistic Abuse	9 (8.6%)	6 (8.1%)	3 (9.7%)	0.7
Financial Abuse	20 (19%)	13 (18%)	7 (23%)	0.6
Seek Help				0.3
No	59 (56%)	39 (53%)	20 (65%)	
Yes (from victims)	44 (42%)	34 (46%)	10 (32%)	
Yes (from bystanders)	2 (1.9%)	1 (1.4%)	1 (3.2%)	
Tactics of Psychological Aggression^3^				
Expressive Aggression	74 (80%)	51 (81%)	23 (79%)	0.9
Coercive Control	38 (41%)	24 (38%)	14 (48%)	0.4
Threat of Physical and Sexual Violence	41 (45%)	17 (27%)	24 (83%)	< 0.001^***^
Exploitation of Vulnerability	10 (11%)	8 (13%)	2 (6.9%)	0.5
Gaslighting	24 (26%)	18 (29%)	6 (21%)	0.4

^1^ n (%)

^2^ Fisher’s exact test; Pearson’s Chi-squared test

^3^ Differences in tactics of psychological aggression by the use of a firearm were examined only among the sample who reported experiencing psychological aggression (*n* = 92, 63, 29 for the total, cases without firearm use, and cases with firearm use, respectively)

*** *p* < .001

For the consequences of firearm-related IPV, the most commonly reported harms were fear (32%), concern for safety (31%), and physical injury (30%). The most frequent protective or help-seeking actions included ending the relationship (67%), moving out (30%), and calling the police (30%). IPV victims who experienced GV reported higher rates of both harms and protective actions than those who did not, although statistically significant differences were observed only for concern for safety and physical injury (Table 6). Of 31 IPV victims of GV, most (73.3%) reported experiencing two or more consequences (median = 3.00).

**Table 6:** Consequences of intimate partner violence and gun violence (*n* = 105)

Characteristic	Overall	Firearm Not Used	Firearm Used	*p*-value^2^
	*N* = 105^1^	*N* = 74^1^	*N* = 31^1^	
Was concerned for safety	33 (31%)	15 (20%)	18 (58%)	< 0.001^***^
Was fearful	34 (32%)	21 (28%)	13 (42%)	0.2
Felt constantly on guard, watchful, or easily startled	4 (3.8%)	2 (2.7%)	2 (6.5%)	0.6
Split up with partner	70 (67%)	47 (64%)	23 (74%)	0.3
Tried hard not to think about it or avoided situations/reminders	0 (0%)	0 (0%)	0 (0%)	
Felt numb or detached from others, activities, or surroundings	4 (3.8%)	2 (2.7%)	2 (6.5%)	0.6
Had nightmares or thought about it when you did not want to	4 (3.8%)	3 (4.1%)	1 (3.2%)	> 0.9
Moved out of home	32 (30%)	19 (26%)	13 (42%)	0.10
Felt guilty or unable to stop blaming yourself or others for the event(s)	17 (16%)	10 (14%)	7 (23%)	0.2
Physical injury	31 (30%)	18 (24%)	13 (42%)	0.071^†^
Missed days of work or school	8 (7.6%)	7 (9.5%)	1 (3.2%)	0.4
Quit work or school	3 (2.9%)	1 (1.4%)	2 (6.5%)	0.2
Called the police	32 (30%)	20 (27%)	12 (39%)	0.2
Sought a protective order	14 (13%)	9 (12%)	5 (16%)	0.5
Reduced or eliminated internet presence	1 (1.0%)	1 (1.4%)	0 (0%)	> 0.9
Sought medical care	8 (7.6%)	5 (6.8%)	3 (9.7%)	0.7
Sought victim’s advocate resources	15 (14%)	9 (12%)	6 (19%)	0.4
Contacted a crisis hotline	2 (1.9%)	2 (2.7%)	0 (0%)	> 0.9
Self-harming	4 (3.8%)	3 (4.1%)	1 (3.2%)	> 0.9
Other	7 (6.7%)	6 (8.1%)	1 (3.2%)	0.7

^1^Fisher’s exact test; Pearson’s Chi-squared test

† *p* < .010, *** *p* < .001

*GV * Gun violence, *IPV * Intimate partner violence

AI, AR, DV, Rel are acronyms for the following subreddits, respectively: */r/AbuseInturrpted*,* /r/abusiverelationships*,* /r/domesticviolence*, and */r/relationships*

## Discussion

This study used Reddit data to examine firearm-related violence perpetrated by intimate partners, focusing on its patterns, behaviors, and consequences. The findings confirmed that Reddit contains valuable information on GV, including specific behaviors and their impact on victims, alongside broader IPV-related discussions. Many IPV victims whose partners used firearms reported being threatened with a firearm. The most common threat involved abusers threatening to shoot the victim, though some also described abusers threatening to shoot themselves (i.e., commit suicide), threatening to harm others, or displaying a firearm as a means of intimidation. GV intensified the impact of IPV, leading to increased psychological aggression, such as threats of physical or sexual violence, as well as heightened safety concerns and physical injury as consequences. While firearm access can also drive or co-occur with other forms of violence, our analysis conceptualizes the firearm primarily as a tool of IPV, used to exert control, intimidate, and inflict physical or psychological harm. This framing situates GV as an instrument through which IPV is enacted, rather than as an independent or incidental phenomenon.

These findings align with previous research using other data sources, such as surveys and interviews. Studies have shown that while firearm access is a key risk factor for IPH [23], non-fatal firearm use is more commonly used to intimidate, control, and threaten intimate partners [24, 25]. For example, Lynch et al. found that verbal threats to shoot victims were the most common firearm-related tactic experienced by IPV victims in domestic violence shelters [24]. Sorenson reported that threats with a firearm occurred more frequently than threats with other weapons [26].

Although less common, some posts in this study described abusers threatening to harm themselves or others. Indeed, Adhia et al. reported that around 22% of U.S. adults who experienced IPV reported that their abuser had threatened to harm someone else more than once within a year [21]. Similarly, Kafka et al. found that IPV accounted for more than 10% of all intentional violent deaths, including not only IPH but also IPV-related suicides and secondary victimization [27]. These findings underscore the broader impact of firearm-related IPV, which extends beyond victims to affect their families and communities, emphasizing the need for societal-level prevention strategies.

Research indicates that firearm use in IPV is more likely to manifest as threats rather than physical violence [25, 26]. Similarly, our study found that while GV did not significantly increase physical violence, it contributed to emotional distress among IPV victims, particularly through threats of physical and sexual violence. Psychological aggression, though common and harmful, is often overlooked or not treated with the same seriousness as physical violence. For instance, legal decisions, such as granting protective orders (POs), are often contingent on visible signs of physical harm [28]. Recognizing the impact of psychological aggression, particularly when firearms are involved, is crucial for improving support and outcomes for victims .

This study also showed that IPV victims who experienced GV were more likely to report heightened concerns for safety and fear. Prior research has similarly documented that fear and safety concerns are common consequences of non-fatal firearm used in IPV [21]. Sorenson reported that victims threatened with a firearm were more likely to feel fear when police arrived than those threatened with other weapons. In our study, fear was conservatively coded, requiring explicit statements from victims, which may have resulted in underreporting. This highlights the challenges in fully capturing the emotional impact of firearm-related IPV.

Lastly, although GV was not strongly linked to increased physical violence in this study, victims who experienced GV reported higher rates of physical injury. This may suggest a history of more severe violence among these victims, even if physical violence was not present during incidents involving firearms. Further research is needed to better understand the relationship between GV and physical injury in IPV cases.

### Implications

This study lays the groundwork for future research using natural language processing and machine learning to automatically identify, in real time, self-reported experiences of firearm-related IPV on social media. Such systems could enable timely connections between victims and appropriate resources at scale. Social media-based large language models have shown promising results in automatically detecting self-reported IPV on Reddit and X [29, 30]. Future studies may explore solutions to support victims present online, like mHealth intervention or chatbot designed in trauma-informed ways, to reach a broader community of IPV and GV victims and improve their accessibility to the existing resources. Simultaneously, researchers must be mindful of potential ethical issues (e.g., unsolicited contact as part of the intervention or tech abuse victimization), which can unintentionally put victims in harm’s way.

From a clinical standpoint, although it was discussed earlier that IPV victims are more willing to share their stories with peers than with healthcare providers, the finding—a higher likelihood of physical injury among IPV victims who experienced GV—indicates a critical role of healthcare providers in identifying signs of IPV, providing resources, and thereby reducing harm or preventing future IPV. Reportedly, however, clinicians are reluctant to engage in gun conversations with their patients, losing opportunities for IPV and GV prevention. Only one in seven (14%) adults say a doctor or other healthcare provider has asked if they own a firearm or if there are firearms in the home [31]. Further, there has been relatively little discussion, let alone agreement, about what healthcare providers should do with firearm-related IPV. Options range from nothing (charting) to informing the victims about laws and procedures by which their abusers can be prevented from purchasing and possessing a firearm [26]. Thus, more efforts should be made to foster the competency of healthcare providers in screening IPV and the involvement of a firearm, consulting, and supporting identified or suspected victims during clinical encounters.

These efforts also extend to other professionals, such as advocates in domestic violence shelters, who play a critical role in preventing harm and fatal outcomes associated with firearm-related IPV. Advocates often face barriers, including fear of offending victims, reluctance to engage in discussions perceived as political (e.g., gun control), and limited knowledge about firearms [32]. Consequently, they may feel uncomfortable initiating conversations about firearms with clients [32]. Understanding victims’ experiences of firearm-related IPV can foster empathy and preparedness among advocates and inform the development of targeted training and tools to enhance their confidence and willingness to address firearm-related IPV in their work.

Moreover, advocates can reimagine their service delivery models to include both in-person and online approaches. Although social media facilitates active exchanges of support and information, these interactions typically occur among peers—past or current victims. There are valuable opportunities for advocates, equipped with expertise and resources, to provide credible information and trauma-informed support to victims seeking help online. For example, Our Wave, a non-profit organization, operates an online platform similar to Reddit that primarily serves individuals affected by sexual violence, where a Sexual Assault Nurse Examiner responds to users’ questions in a trauma-informed manner [33]. This model offers a promising example of expanding service reach from traditional in-person settings to online spaces.

Despite the potential lethality of firearm-related IPV, victims were not necessarily prone to call the police or seek a PO. Previous experiences with racial or social injustice can further complicate help-seeking, particularly among victims from marginalized communities [34]. This underscores the need for ongoing police efforts to recognize and address bias, treat all victims with respect, investigate complaints thoroughly and effectively, refer victims to appropriate services, properly identify perpetrators, hold officers accountable for misconduct (e.g., domestic violence), and review and act upon data [35]. When responding to IPV or domestic violence scenes, police officers often focus on visible signs of physical violence [36], even though firearm-related IPV may inflict more psychological than physical harm. Greater emphasis should be placed on understanding how to enforce the law when responding to non-physical forms of violence [36]. Training should also enhance officers’ awareness of various tactics of psychological aggression, including coercive control and threat of physical and sexual violence, to strengthen their ability to respond effectively to the full spectrum of firearm-related IPV incidents [36].

From a policy standpoint, prohibiting the purchase and possession of firearms as a condition of a PO remains one of the most effective means to prevent future IPV and protect victims from potentially fatal outcomes [37]. However, Lynch et al. reported that fewer than half of IPV victims who requested a PO were aware that their partner was prohibited from possessing a firearm during the order, and only about 10% reported that a judge had ordered their partner to surrender firearms [24]. However, when judges did issue such orders, victims were more likely to report that all firearms were either surrendered by the abuser or confiscated by law enforcement [38]. Although officers assess firearm involvement during IPV or domestic violence incidents, their authority to remove firearms from the scene, whether in plain view, used, or threatened to be used, depends on state laws [39]. Currently, only 20 states authorize law enforcement to remove firearms from the scene of domestic violence [39]. These findings underscore the need for laws restricting firearm access among IPV abusers and authorizing officers to remove firearms to be clearly drafted and consistently enforced across states, along with targeted interventions to raise victims’ awareness that POs can limit their abusers’ firearm access. Such interventions may be especially valuable for those who avoid seeking formal help through the criminal justice system [24]. A greater understanding of the protective benefits of POs could help mitigate hesitancy rooted in distrust or systemic barriers.

### Limitations

This study has limitations. First, we likely missed OPs with meaningful information related to firearm-related IPV by limiting the data only to English-written OPs from four subreddits; the keywords used to identify firearm-related OPs were also not exhaustive. Second, given the characteristics of social media users and the small sample size, the findings may not be generalizable. IPV victims on Reddit are a selected group of victims who have access to devices, the Internet, and a relatively safe environment to use social media. Victims under their partner’s strict control, including tech abuse, may not have security in their access to private use of social media and may have been underrepresented in the collected data. Therefore, results should be interpreted as exploratory and hypothesis-generating rather than conclusive. Third, as mentioned earlier, because GV occurred as a part of IPV, the needs and consequences of GV could not be separated from those of IPV in general. Fourth, users’ sociodemographic (e.g., age and gender) and geolocation data could not be easily collected unless users voluntarily shared that information, although such data are critical to mapping the distribution of firearm-related IPV to develop prevention strategies. Lastly, responses to the OPs were not analyzed, which would have been another useful source of data to characterize firearm-related IPV and illuminate the nature of interactions between the users.

Nevertheless, this study is the first to examine firearm-related IPV using survivor narratives publicly available on Reddit, offering valuable insights into this critical public health issue and highlighting the potential of social media data to inform violence prevention and intervention efforts.

## Conclusions

This study showed a landscape of firearm-related IPV on Reddit, demonstrating the value of Reddit text data for understanding and preventing firearm-related IPV that can escalate to fatal outcomes. Despite ongoing efforts, there remains a lack of valid and reliable data on non-fatal firearm use in IPV. The gap exists not only because such experiences are deeply sensitive, but also because many victims live under coercion and fear—isolated and forced to endure IPV and GV out of public view. In light of this, Reddit text data are a viable alternative to the widely used data collection methods (e.g., surveys) since their findings are in general agreement with the existing evidence, and Reddit data provide completely user-centered narratives enabling us to have deeper understanding of victims’ violence experiences and circumstances that may not be easily collected by other sources. Researchers, practitioners, and policymakers can harness Reddit data to inform and develop preventive strategies relevant to victims of IPV and GV and, hence, more impactful in reducing IPV and GV in the country.

## Data Availability

The data analyzed were collected from publicly available Reddit posts; no identifiable private information was used. The data source can be accessed at [https://www.reddit.com]. Although the authors do not own the data, the datasets used and/or analyzed during the current study are available from the corresponding author upon reasonable request.

## Abbreviations

AI: r/AbuseInturrpted
API: Application Programming Interface
AR: r/abusiverelationships
CDC: Centers for Disease Control and Prevention
DV: r/domesticviolence
GV: Gun Violence
IPH: Intimate Partner Homicide
IPV: Intimate Partner Violence
mHealth: Mobile Health
NISVS: National Intimate Partner and Sexual Violence Survey
OP: Original Post
PI: Principal Investigator
PO: Protective Order
PRAW: Python Reddit Application Programming Interface Wrapper
RA: Research Assistant
Rel: r/relationships
U.S.: United States
